# Supplementary material for: Characterization of a strain-specific CD-1 reference genome reveals potential inter- and intra-strain functional variability
Source: BMC Genomics. 2023 Aug 3;24:437. doi: 10.1186/s12864-023-09523-x (PMC10401811; doi:10.1186/s12864-023-09523-x)
Supplement: Supplementary file 1 — Additional file 1. [file 12864_2023_9523_MOESM1_ESM.docx]

**Figure S1**

1.
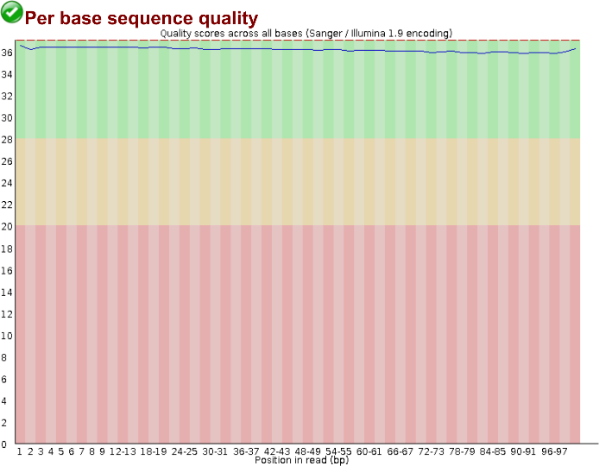

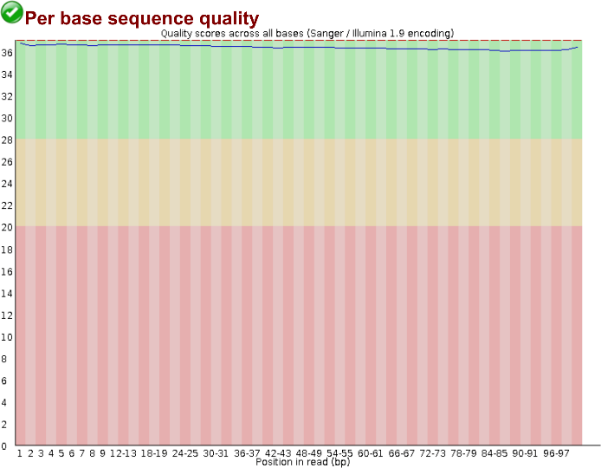
 **CD1-brain-M-2M-DNA-4**
2. **CD1-brain-M-3M-DNA-1**

**
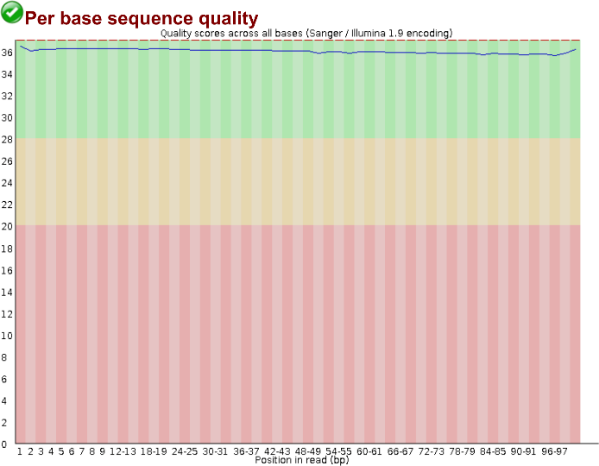

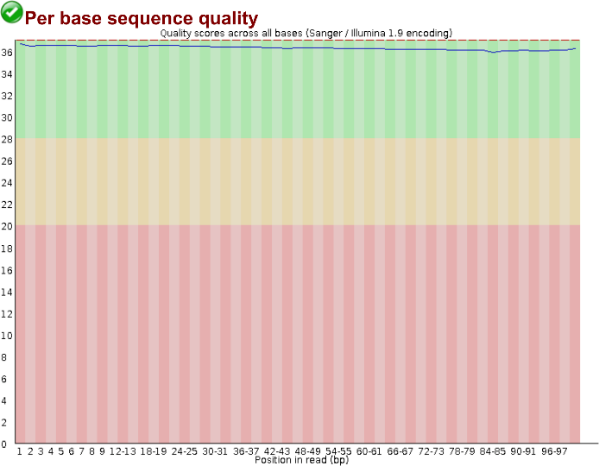
**

1. **CD1-brain-M-4M-DNA-2**

**
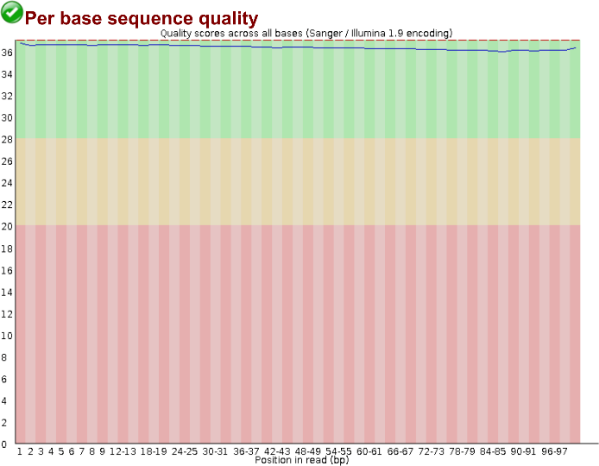

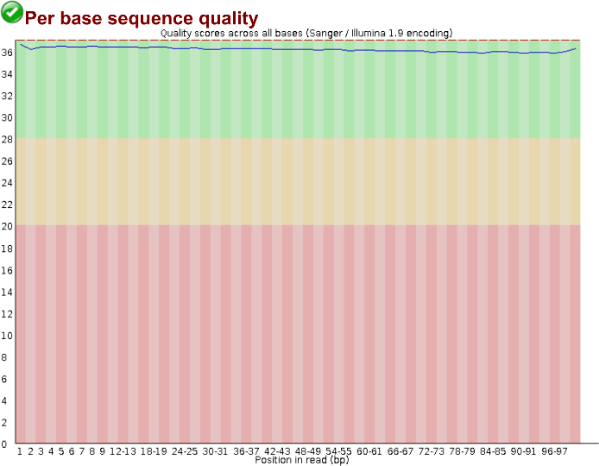
**

**Figure S1 (Cont.)**


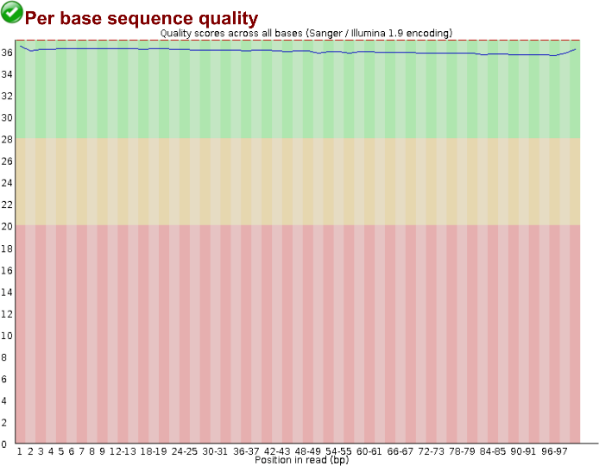

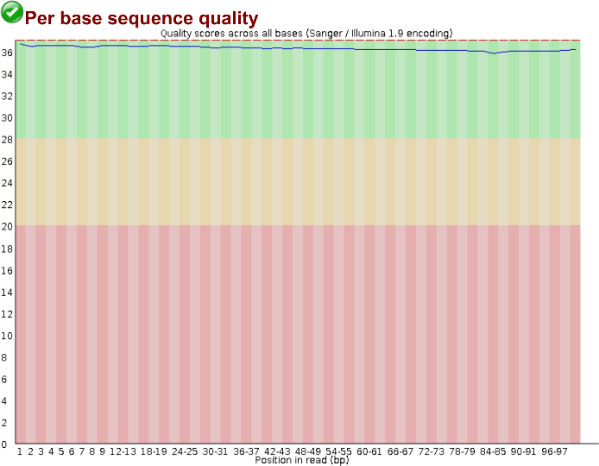
**D. CD1-brain-M-4M-DNA-3**

**E. CD1-brain-M-5M-DNA-5**


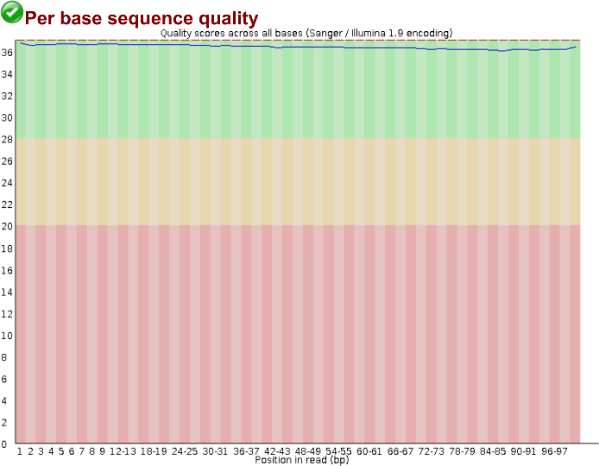

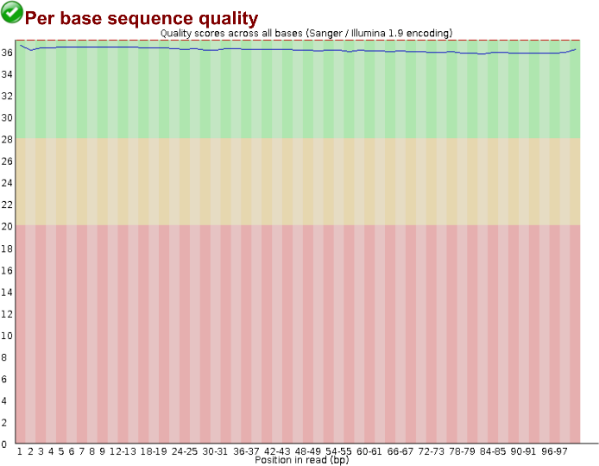


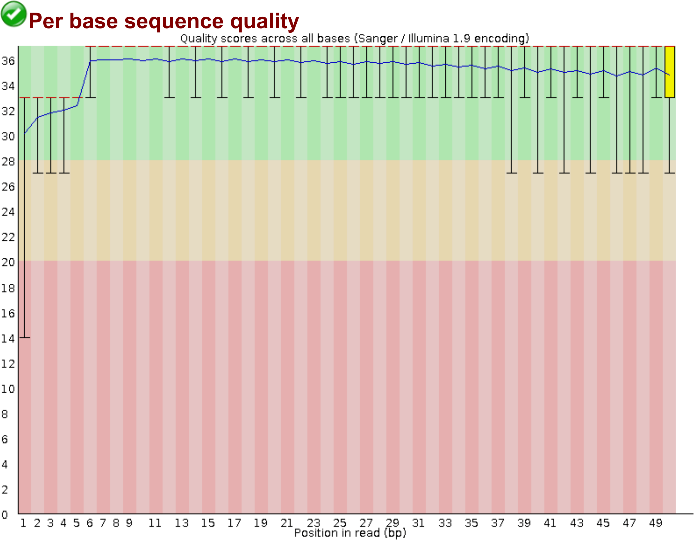
**F. Cont-Adipose-ATACseq-Rep1**


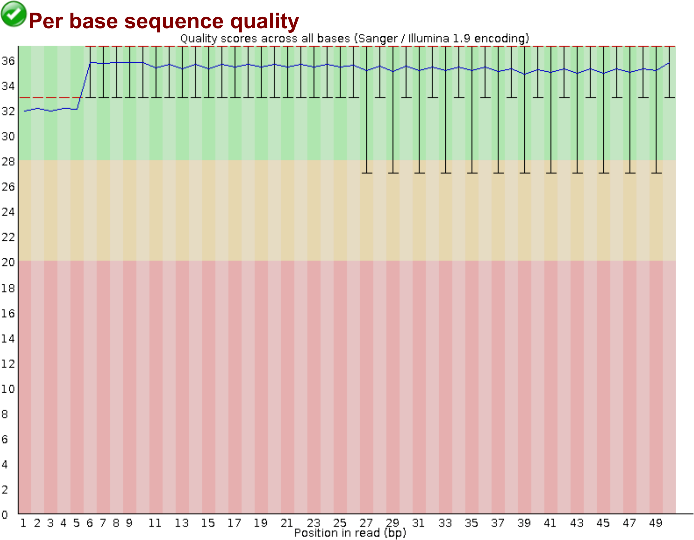


**Figure S1 (Cont.)**


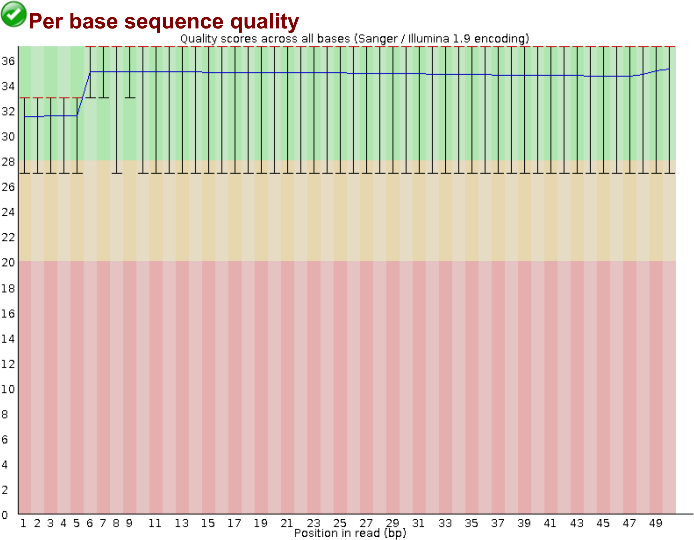

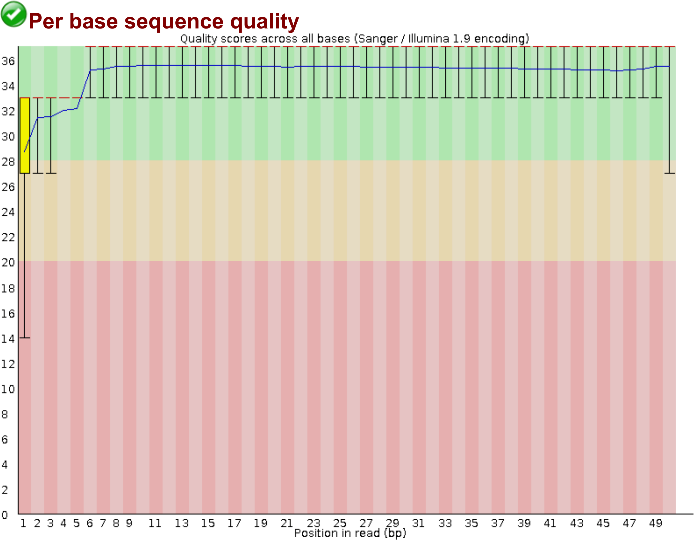
**G. Cont-Adipose-ATACseq-Rep2**


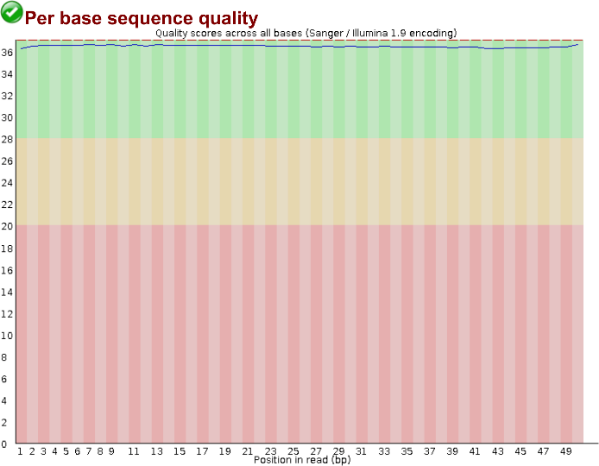

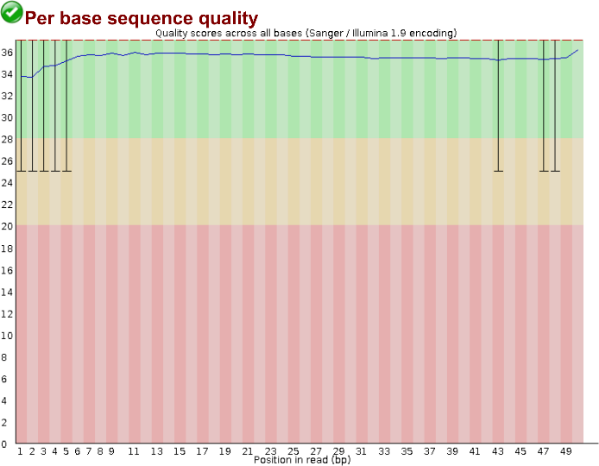
**H. BPAF4-Adipose-ATACseq-Rep1**

**I. BPAF4-Adipose-ATACseq-Rep2**


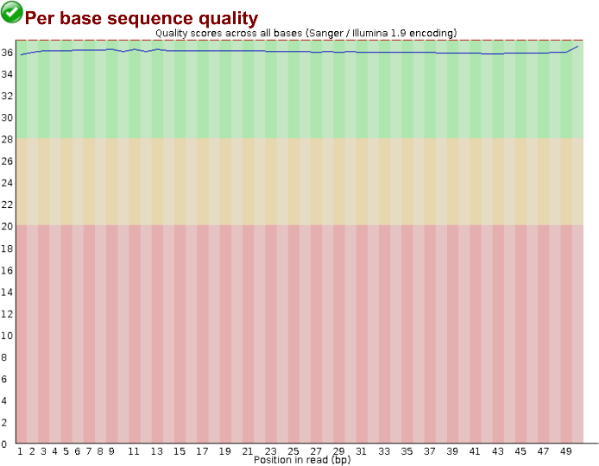

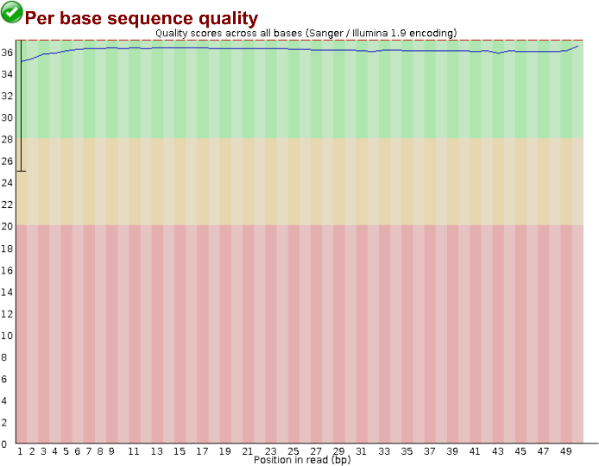


**Figure S1 (Cont.)**


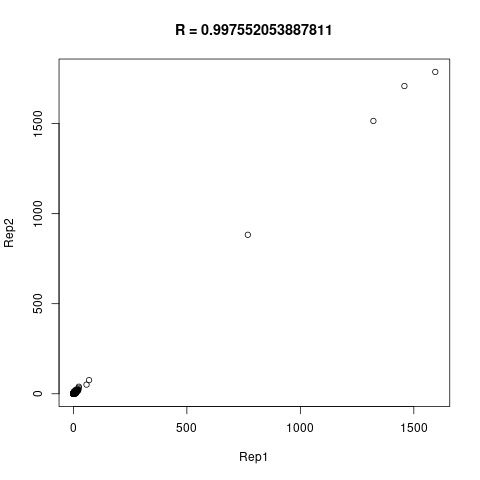

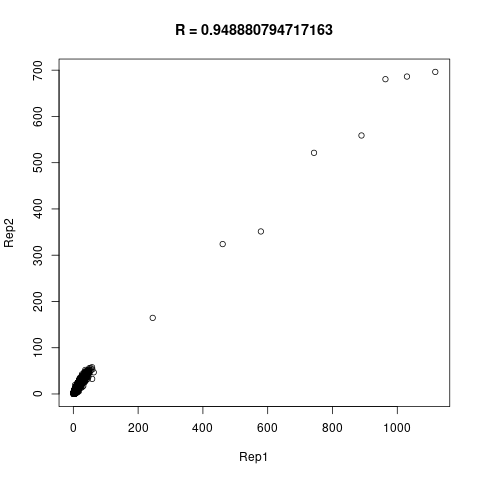
**J.** BPA Control

**K. Validation SNPs**

**
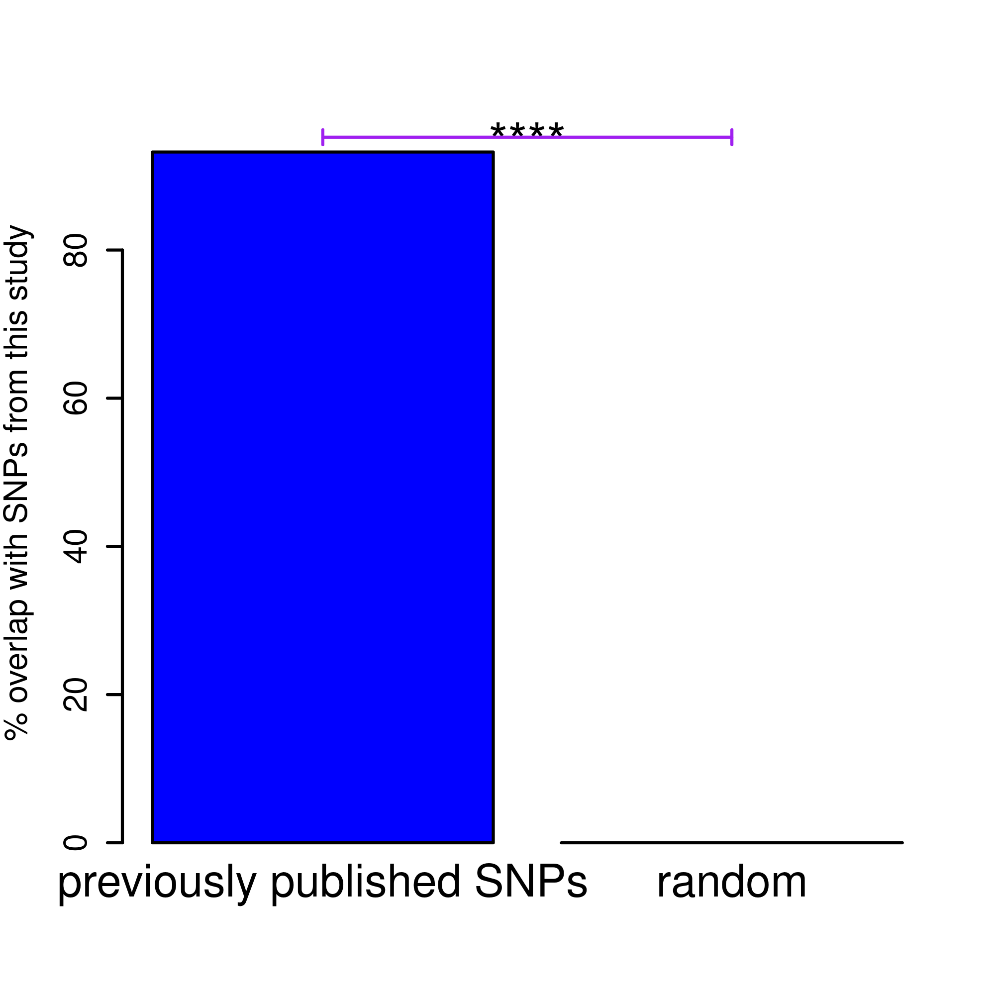
**

**Figure S1 (Cont.)**

**L. Non-Uniform SNPs Non-Uniform SNPs**

**
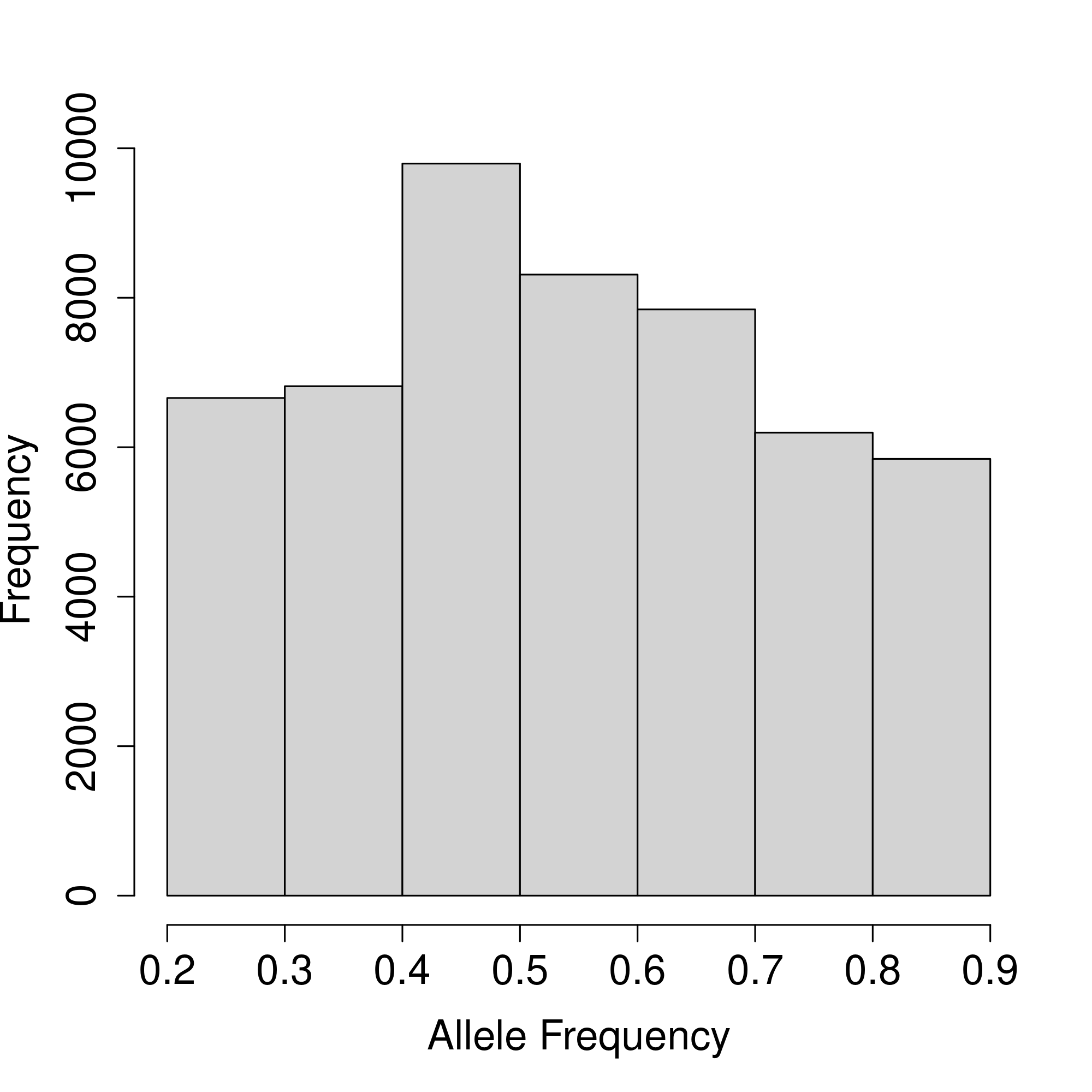

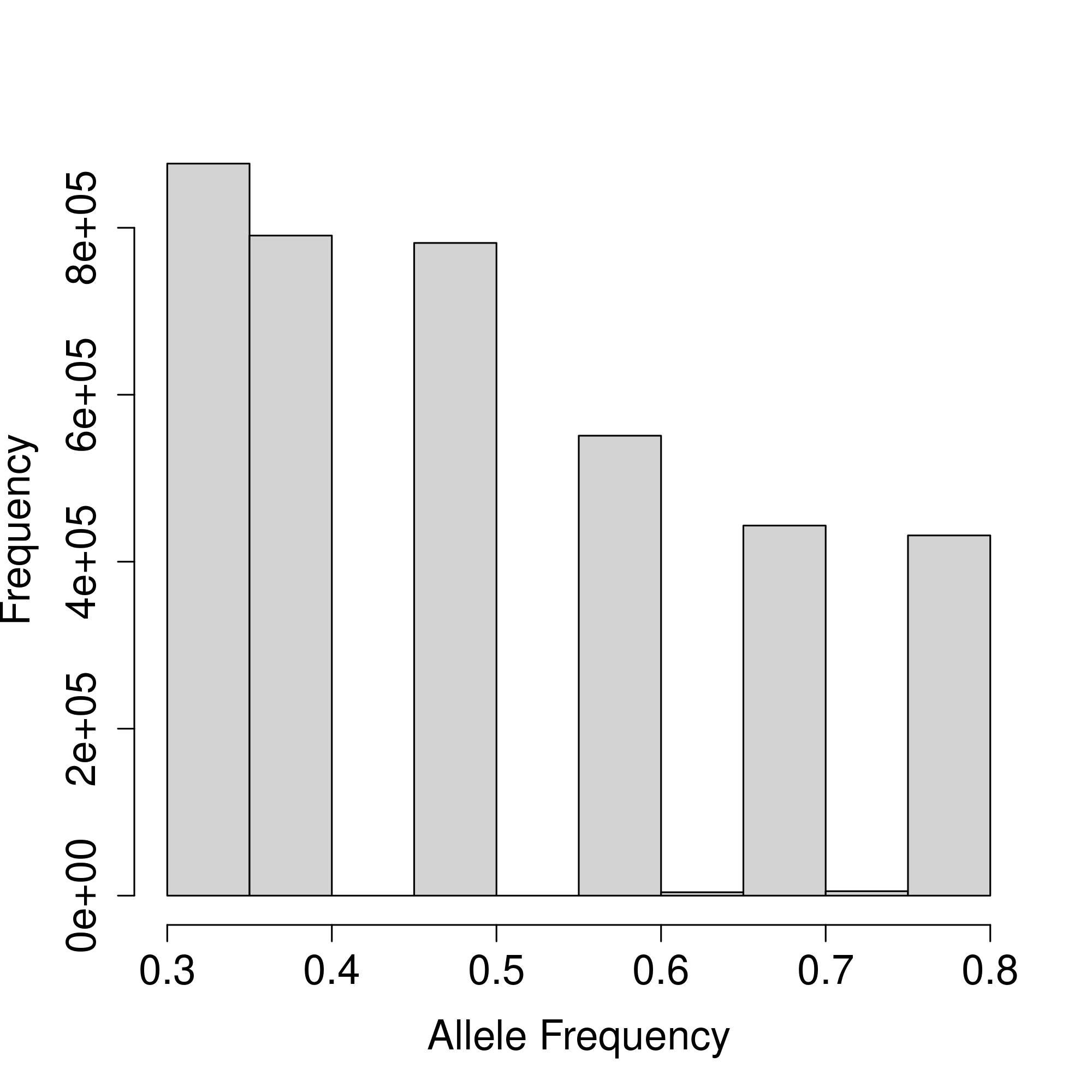
 1 alt. allele >1 alt. allele**

**Uniform Indels Uniform SNPs**

**1 alt. allele 1 alt. allele**

**
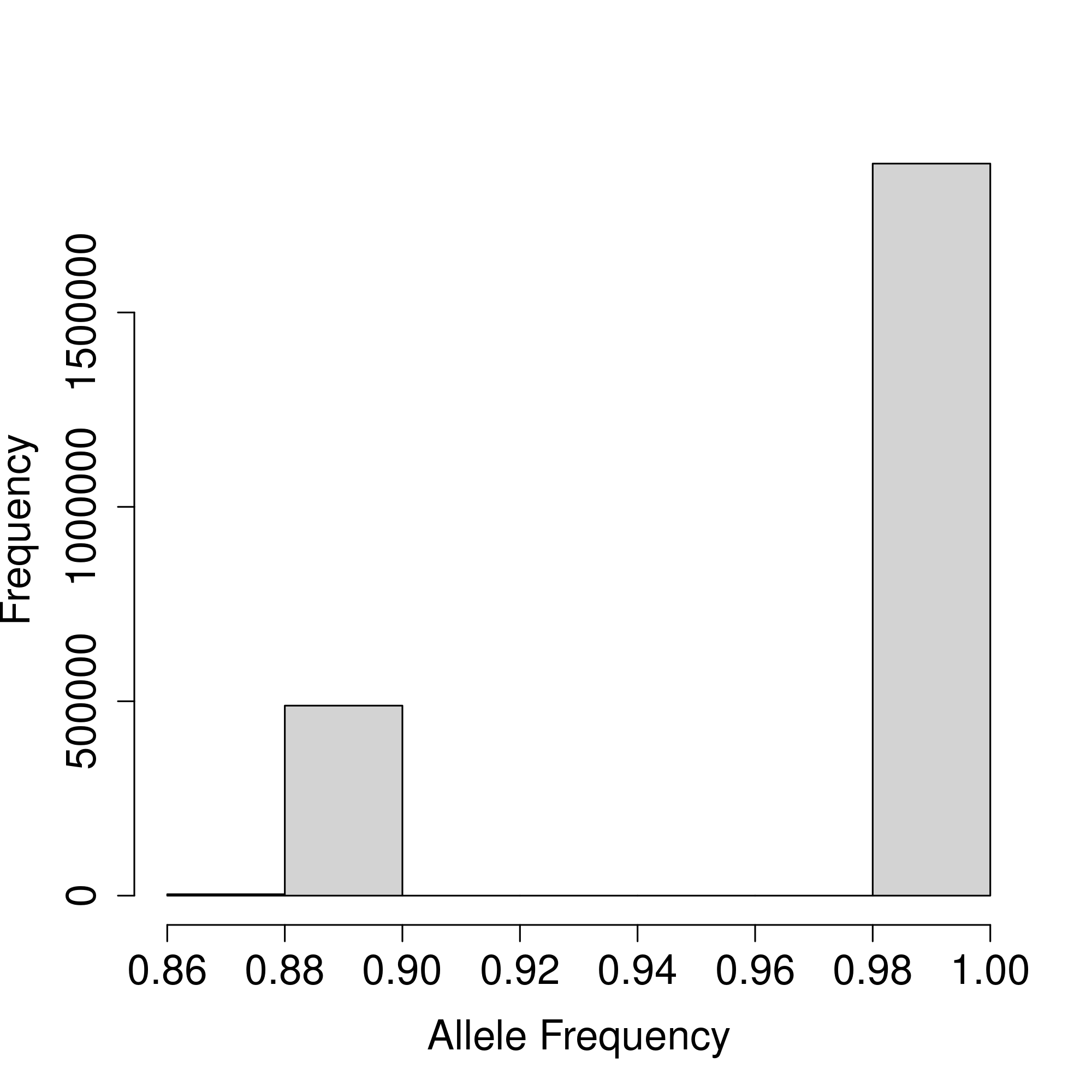

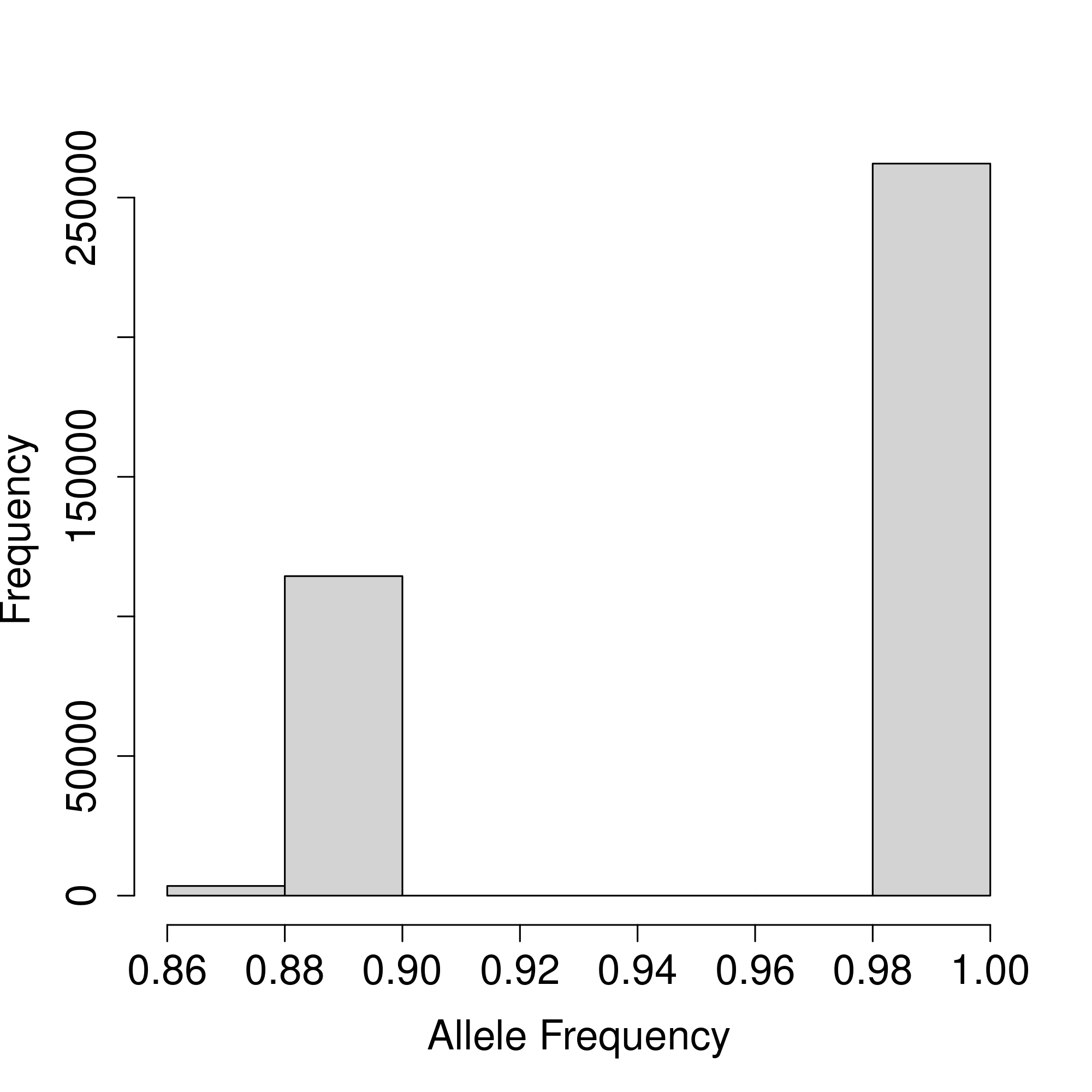
**

**Non-Uniform Indels Non-Uniform Indels**

**1 alt. allele >1 alt. allele**

**
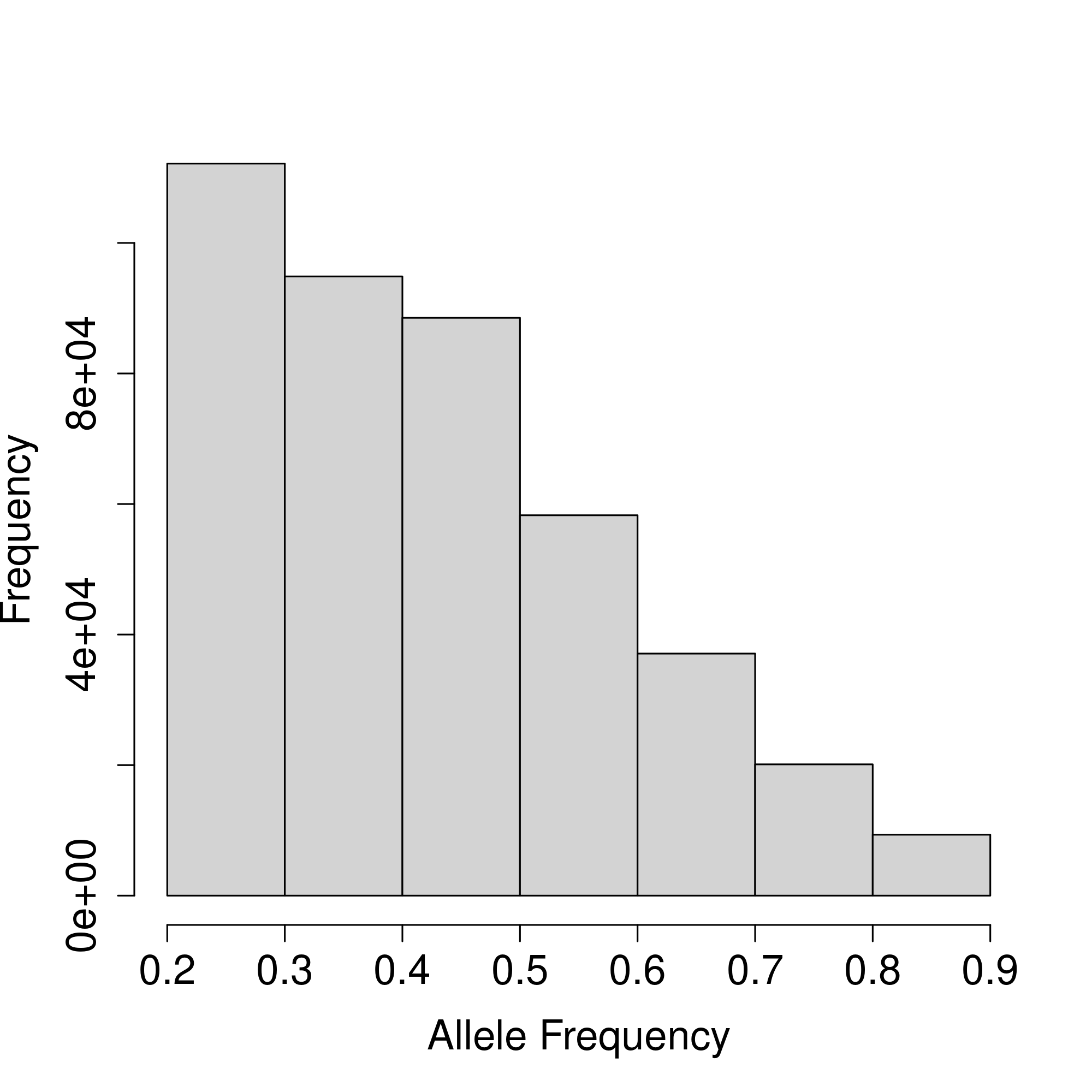

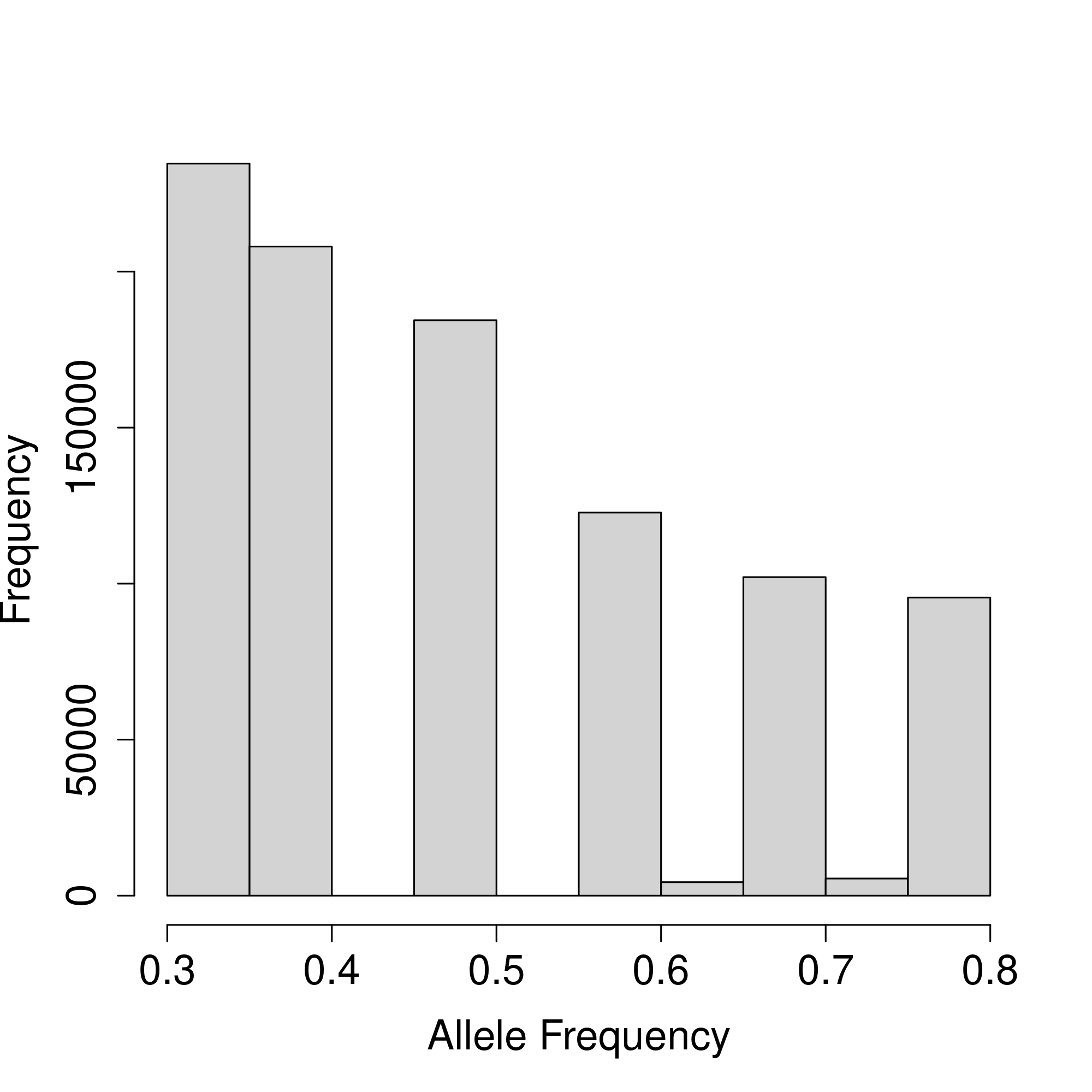
**

**Figure S1 (Cont.)**

**M.**

**
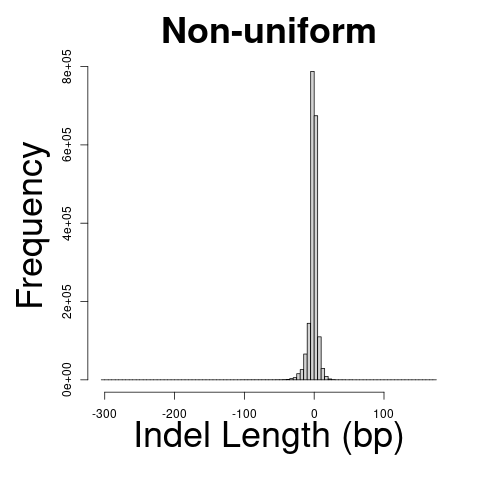

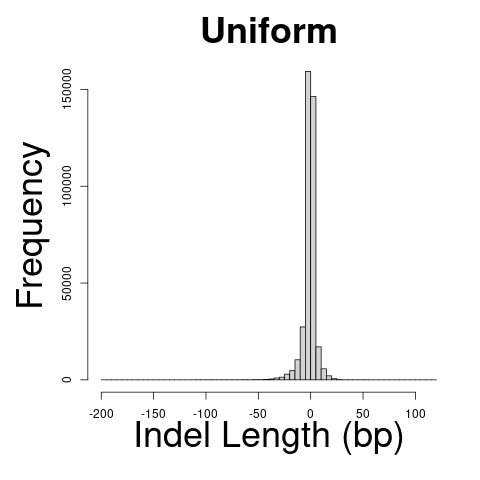
**

**
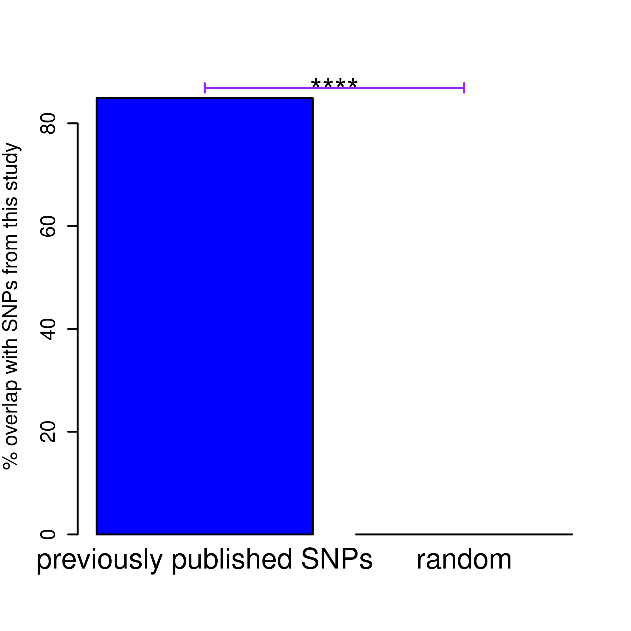

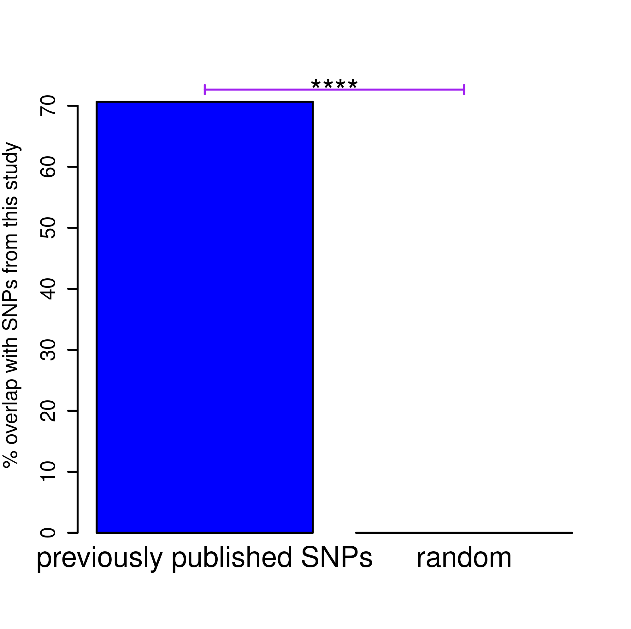
N. Non-uniform Unifor****m**

**Figure S1 (Cont.)**

**O.**

**
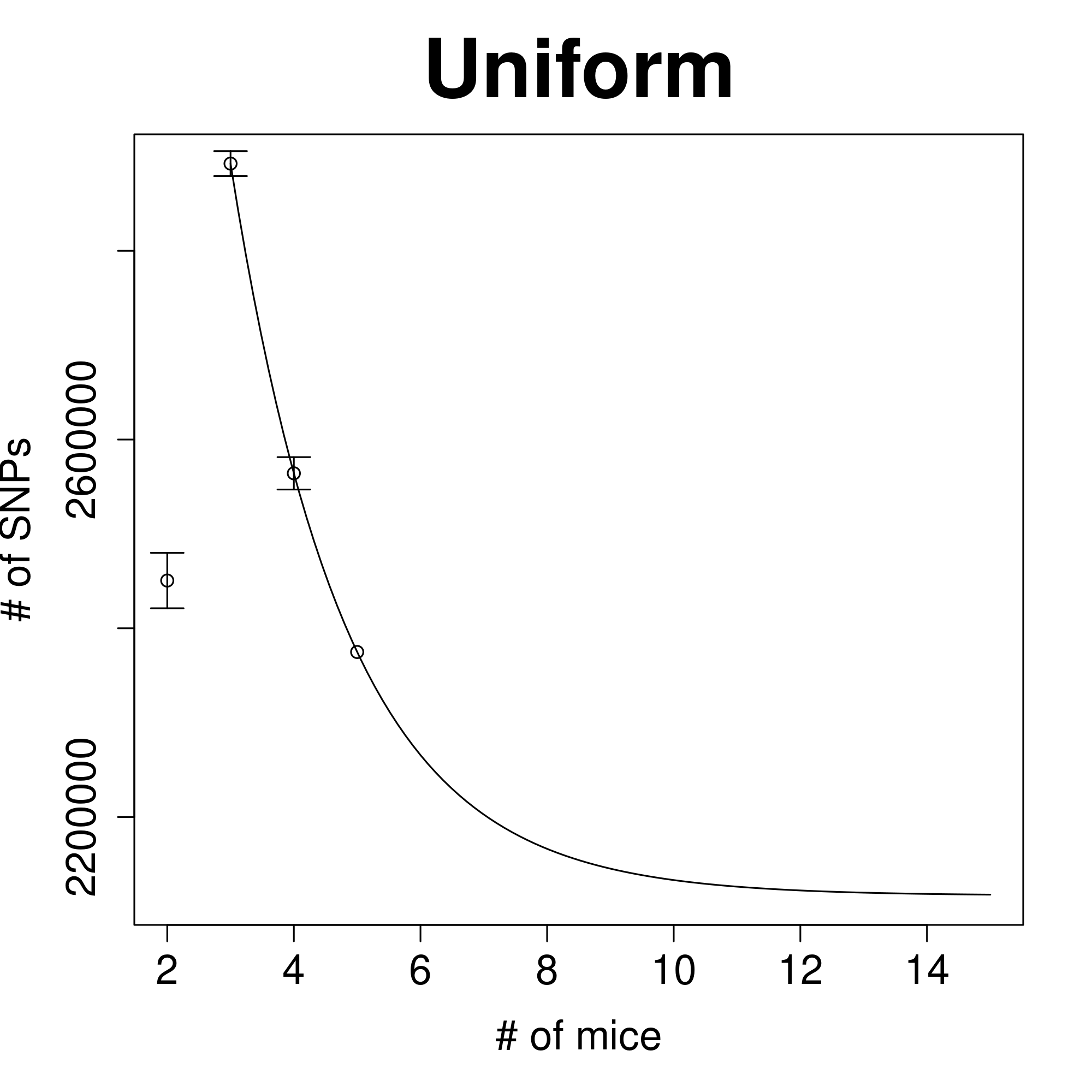

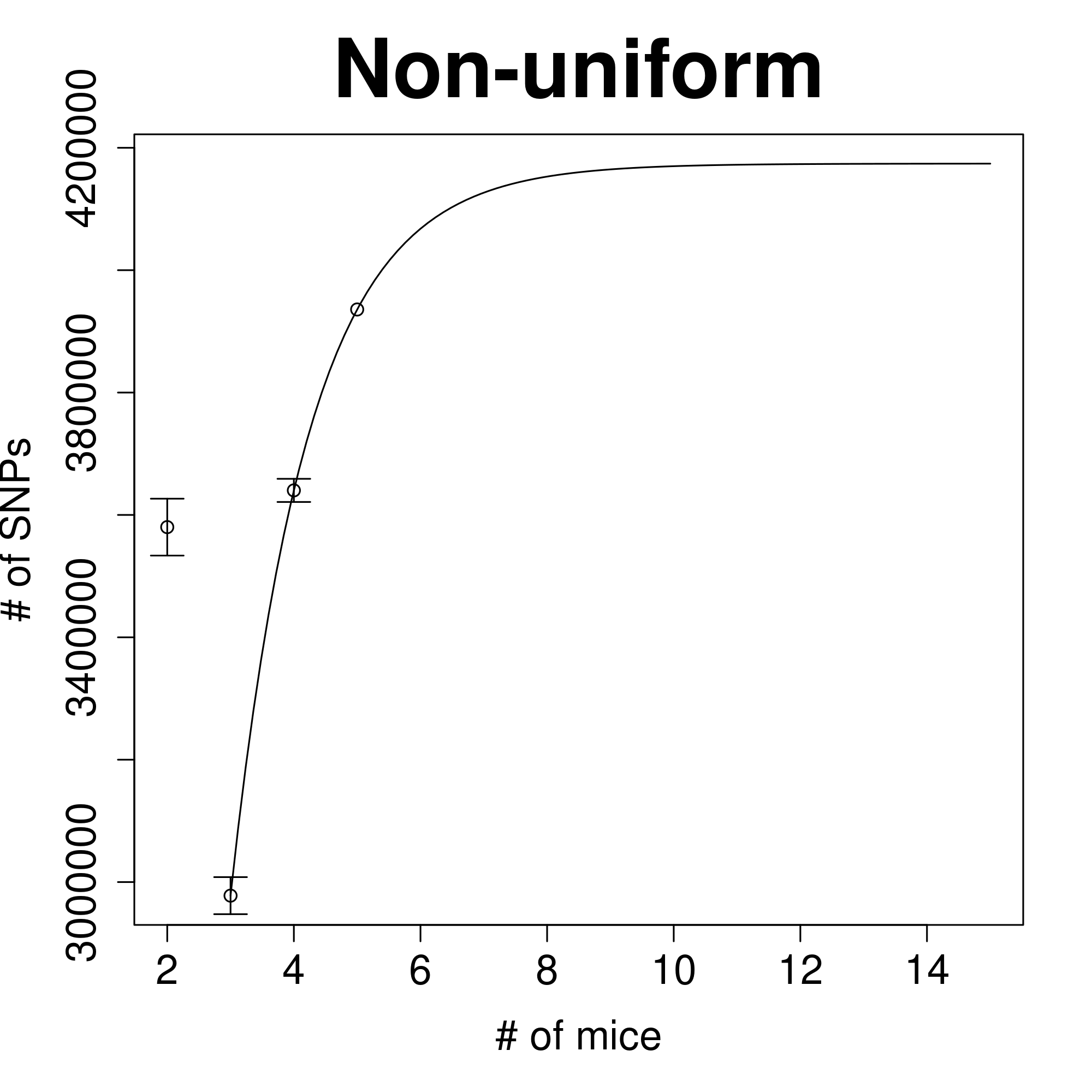
**

**Figure S1. Validation of sequencing data generated in this study. A-E** Plots showing the distribution of base quality scores at each position along the left and right read pair (left and right plots, respectively) for the indicated Cd1 gDNA sample. **F-I** Same as A-E, except for the indicated ATAC-seq sample. **J** Scatterplots comparing RPKM values of the two replicates of the indicated ATAC-seq sample generated in this study (BPA or Control). Pearson’s correlation coefficient is shown above each plot. **K** Barplot showing the percent overlap of the combined set of uniform and non-uniform, previously published SNPs that overlap with our SNPs before removal of low abundance and low frequency SNPs, and comparing that to the percent overlap of randomly shuffled regions with our SNPs before removal of low abundance and low frequency SNPs. **L** Histograms of the frequencies of the most abundant alternative allele for the indicated variant type. 1 alt. allele = variants with only 1 alternative allele that differs from the reference (mm10) allele; >1 alt. allele = variants with more than one alternative allele that

**Figure S1 (Cont.)**

differs from the reference (mm10) allele. Categories not shown do not have any variants. **M** Histograms of the distribution of non-uniform and uniform indel lengths (left and right plot, respectively). Positive values indicate insertions while negative values indicate deletions. **N** Same as K, except using just non-uniform SNPs (left panel), and just uniform SNPs (right panel). **O** Plots showing the number of non-uniform and uniform SNPs acquired upon down-sampling to the indicated number of mice. Down-sampled points are averaged over all possible subsets, and the mean±S.E.M. are shown. Also shown are asymptotic curves fitted using nonlinear least squares regression. P-values in this figure were calculated by Fisher’s exact test, with cutoffs shown as follows: * p < .01 ; ** p < .001 ; *** p < .00001 ; **** p < .0000000001.

**Figure S2**

**
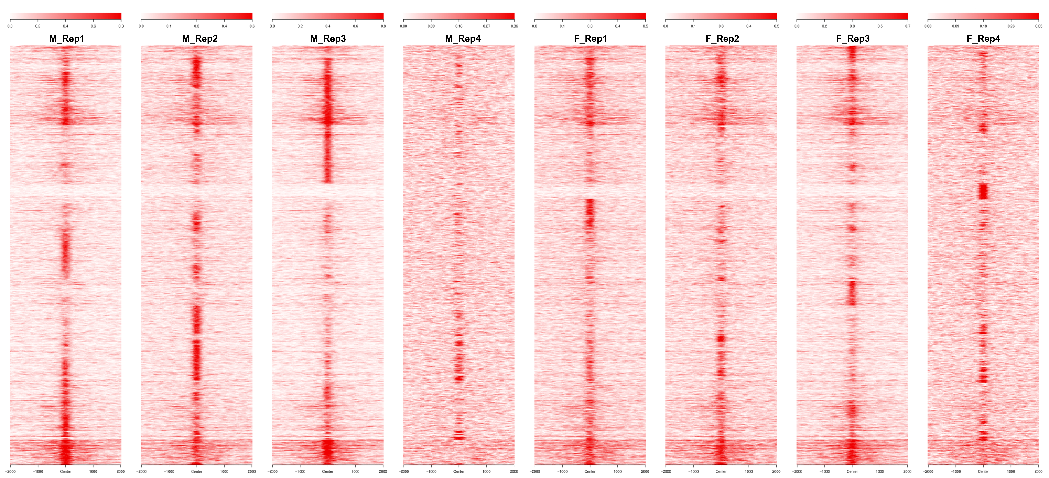
A.**

N=9602


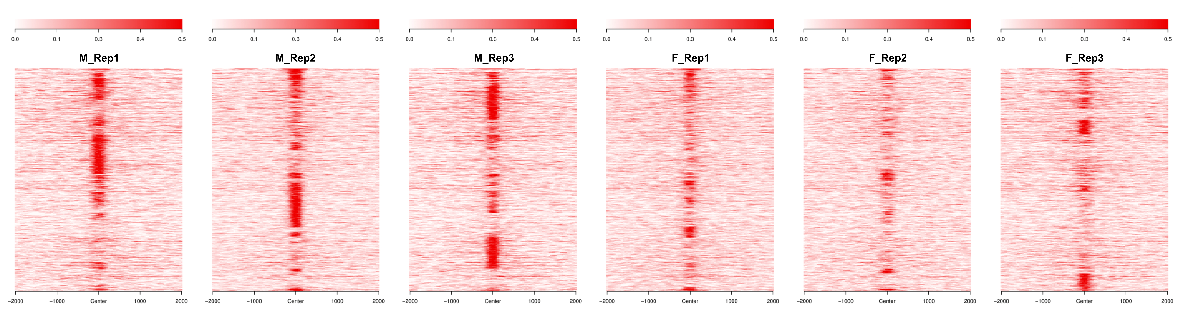
**B.**

N=3387

**Figure S2 (Cont.)**

**
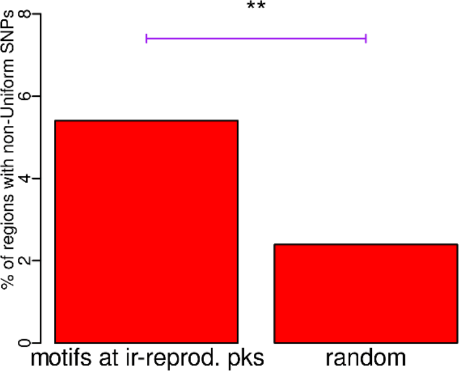

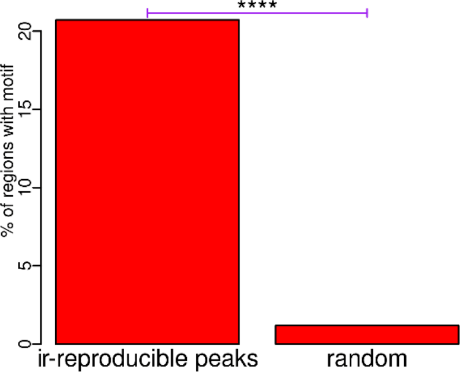
C. D.**

motifs at irreprod. pks random

irreproducible peaks random

**_
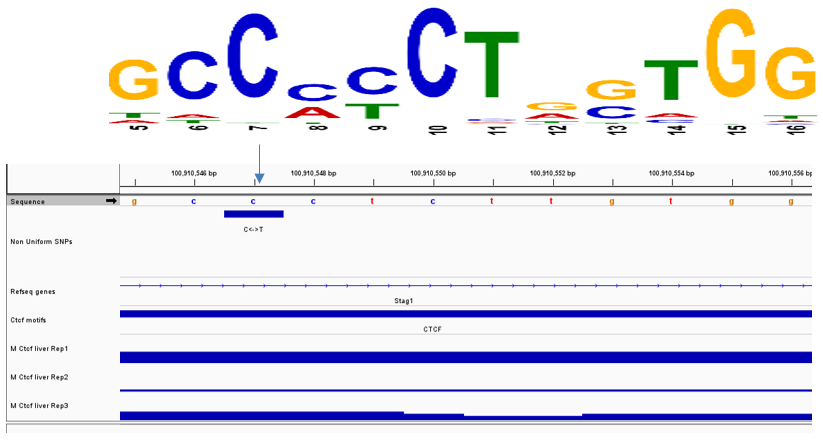
_E.**

**_
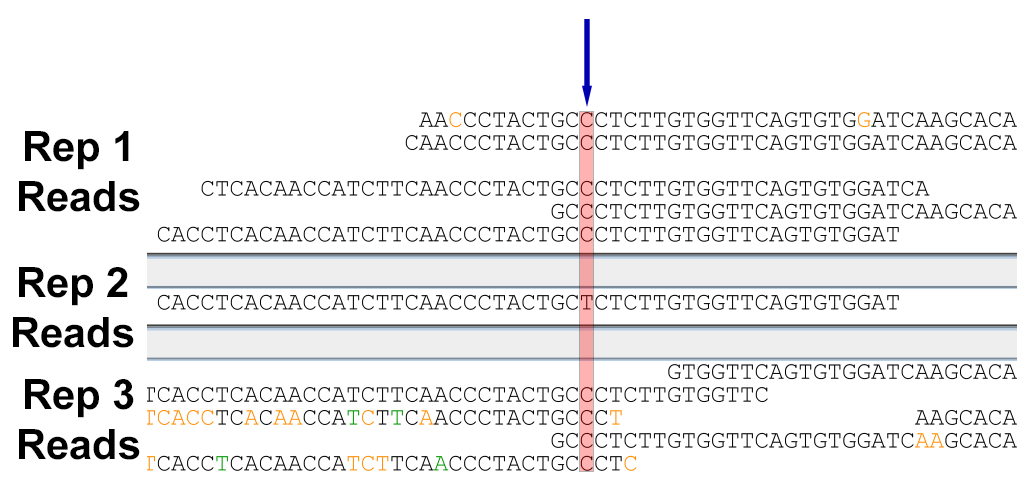
_F.**

**Figure S2 (Cont.)**

**
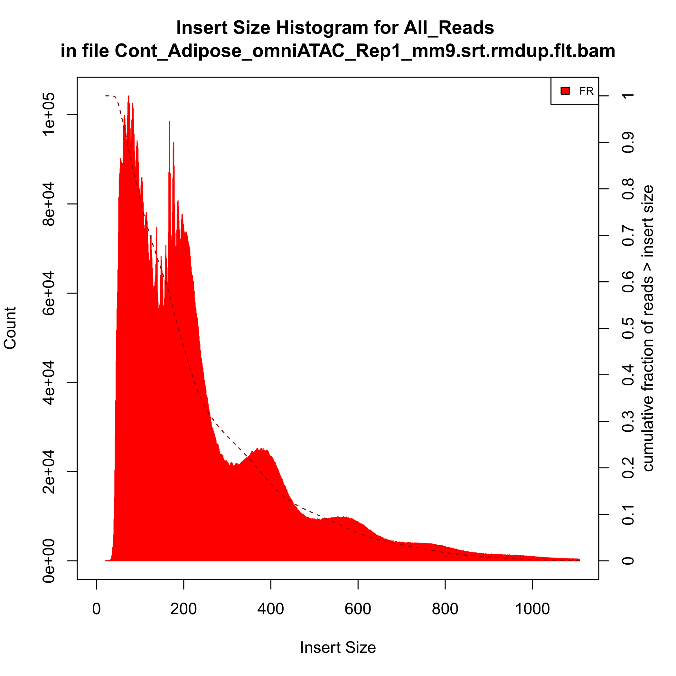

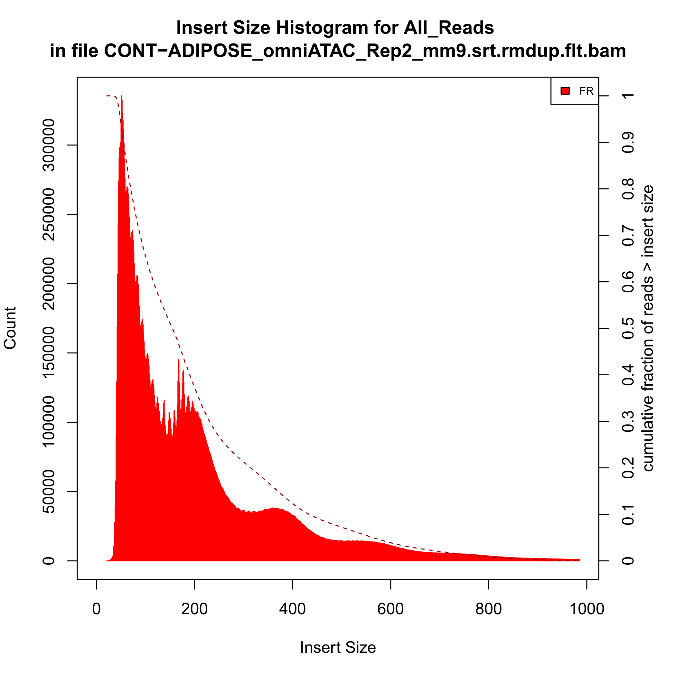
G.**

**
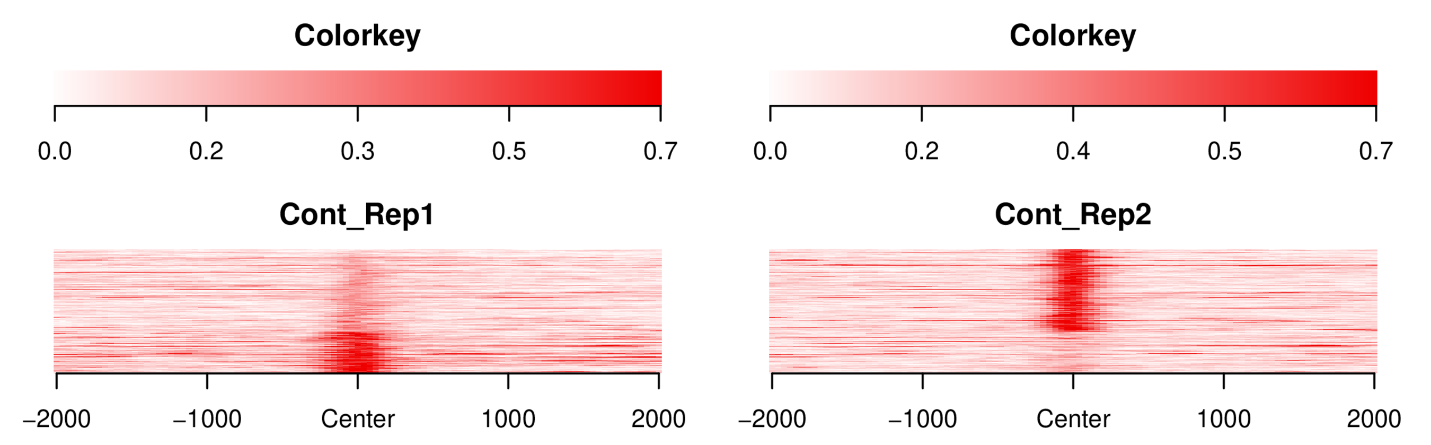
H.**

N=521

**Figure S2. Related to Figure 1. A** Heatmaps showing the enrichment of Ctcf ChIP-seq signal at irreproducible peaks when all four replicates from both male and female liver samples were considered. **B** Same as **A,** except in this case, only considering replicates 1-3 of both the male and female samples. **C** Barplots comparing the percentage of CTCF irreproducible peaks (with

**Figure S2 (Cont.)**

replicates 1-3 of both male and female samples considered) with a called CTCF motif near the summit, to the ratio of randomly shuffled regions that contain a CTCF motif. **D** Barplots comparing the percentage of CTCF motifs at irreproducible peaks (with replicates 1-3 of both male and female samples considered) that overlap with a non-uniform SNP to the percentage of randomly shuffled regions that overlap a non-uniform SNP. Only motifs within 25 bp of a summit were considered. **E** Genome browser image of Reads per million (RPM)-normalized CTCF ChIP-seq coverage around a non-uniform SNP that occurs at a highly conserved C in a called CTCF motif. The reverse-compliment CTCF motif is pictured at top. **F** The sequenced CTCF ChIP-seq reads from the indicated replicate that map to the non-uniform SNP displayed in Figure S1E. Nucleotides are colored according to their phred sequencing quality score as follows: black for 30 or above, orange < 30, green < 20, blue < 10. The non-uniform SNP is highlighted. **G** Histograms of the fragment length distribution for the two replicates of adipose ATAC-seq in control, CD-1 mice. **H** Heatmaps showing enrichment of ATAC-seq signal at irreproducible ATAC-seq peaks. P-values in this figure were calculated by Fisher’s exact test, with cutoffs shown as follows: * p < .01 ; ** p < .001 ; *** p < .00001 ; **** p < .0000000001.

**Figure S3**

**A.
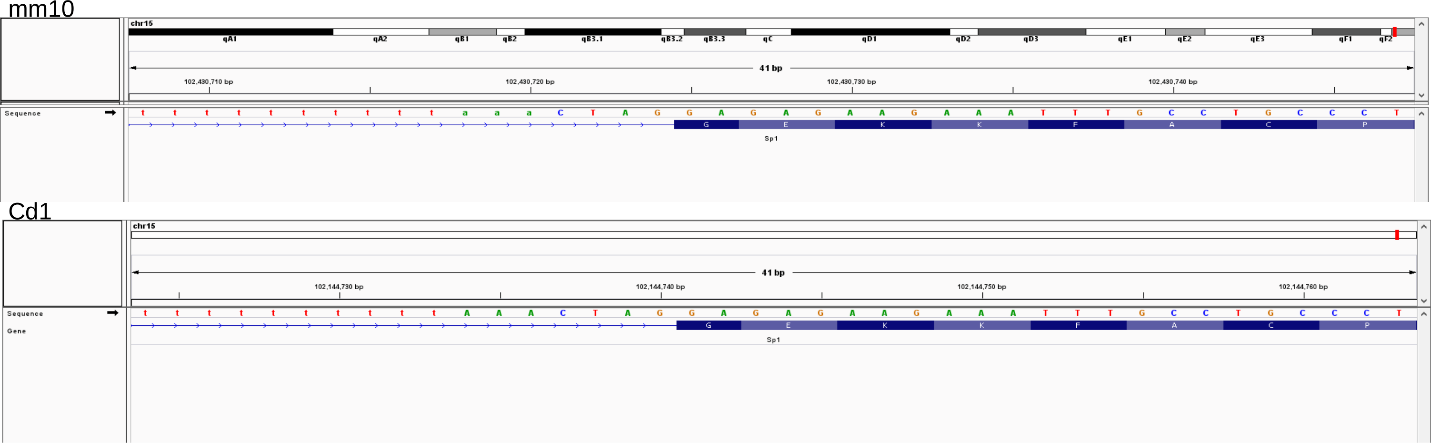
**

**B.
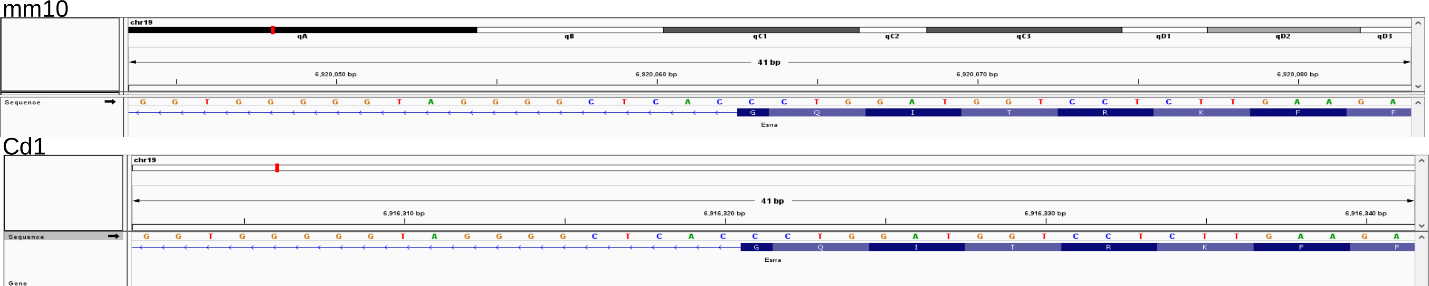
**

**
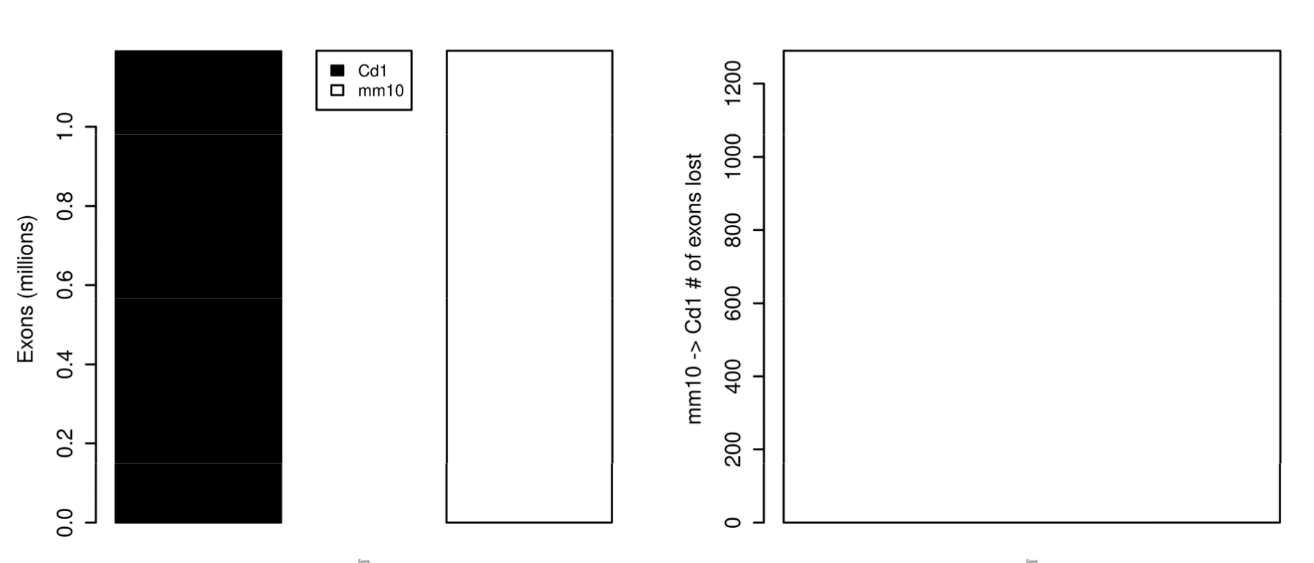
C.**

**Figure S3 (Cont.)**

**
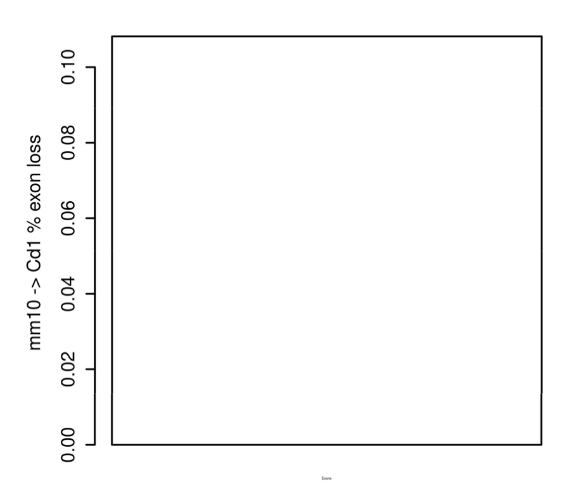
D.**

**
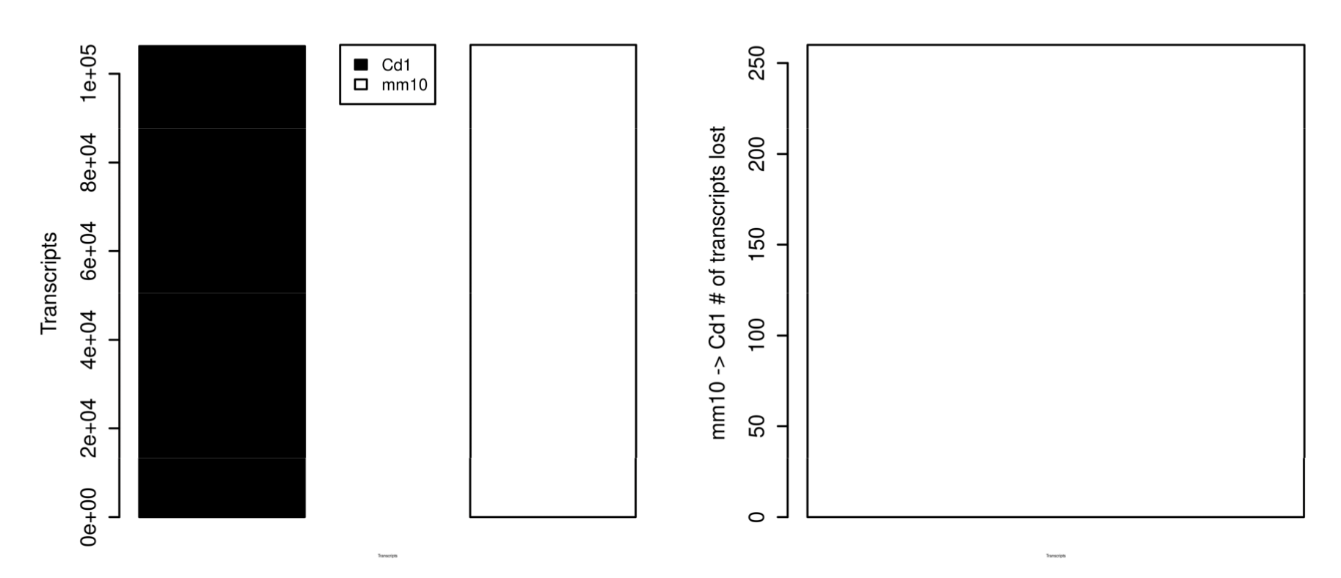
E.**

**
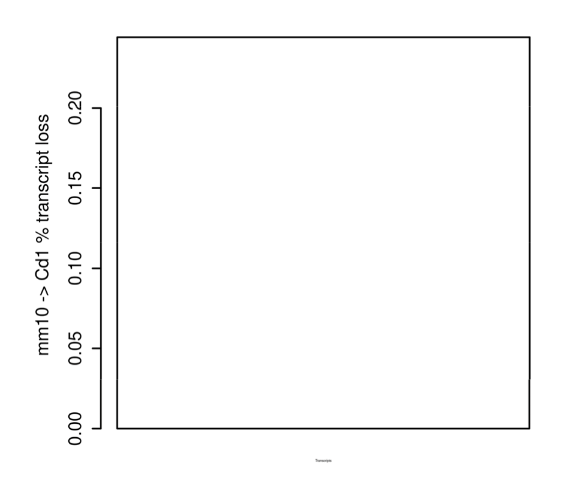
F.**

**Figure S3 (Cont.)**

**Figure S3. Validation of the newly generated CD-1 gene annotations. A** Genome browser views in mm10 coordinates (top) and Cd1 coordinates (bottom) around an intron-exon junction of the Sp1 gene. Both the mm10 and Cd1 nucleotide sequences at this region are displayed for comparison. **B** Same as **A**, except for an intron-exon junction of the Esrra gene. **C** Barplots comparing the number of annotated exons in mm10 to the number of exons successfully converted to CD-1 (left panel), and the number of exons lost in the CD-1 gene annotation (right panel). **D** The percentage of annotated mm10 exons lost when converting to CD-1. **E** Barplots comparing the number of annotated transcripts in mm10 to the number of transcripts successfully converted to CD-1 (left panel), and the number of transcripts lost in the CD-1 gene annotation (right panel). **F** The percentage of annotated mm10 transcripts lost when converting to CD-1.

**Figure S4**

**
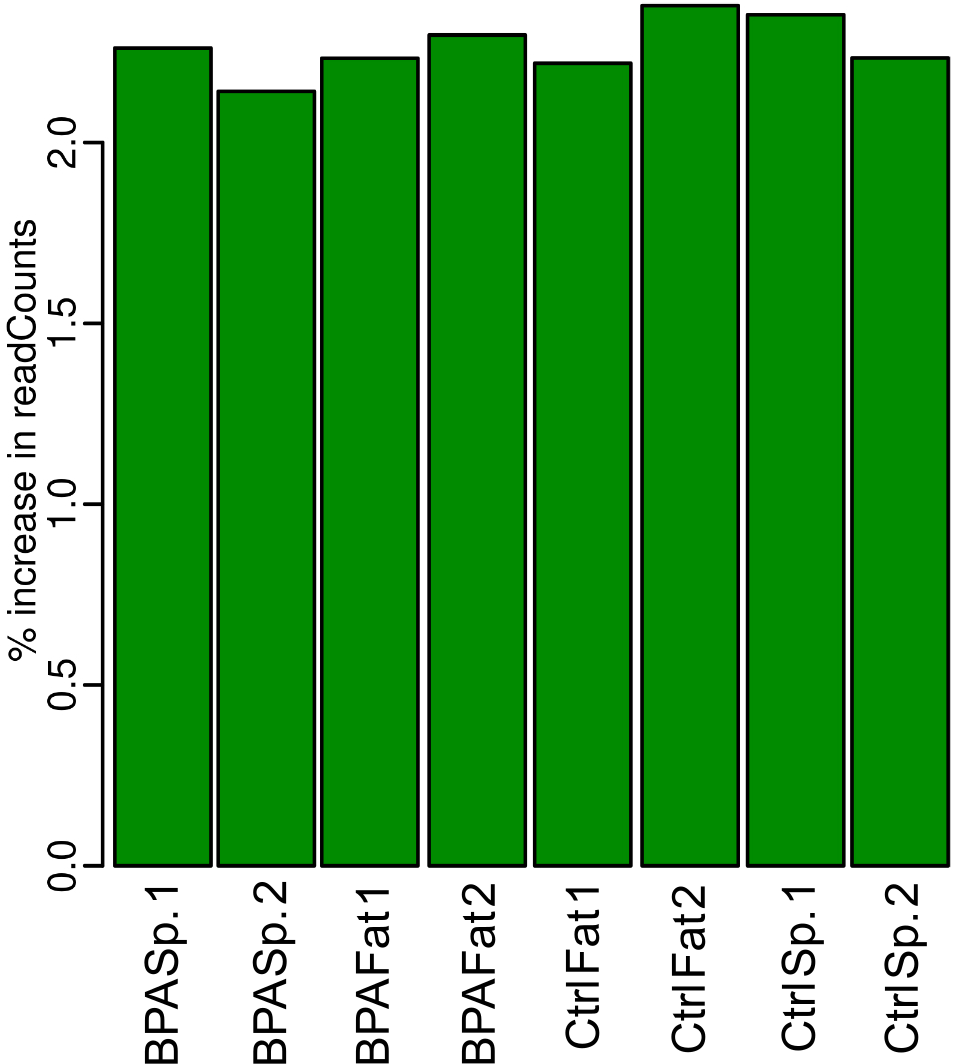
A.**

**
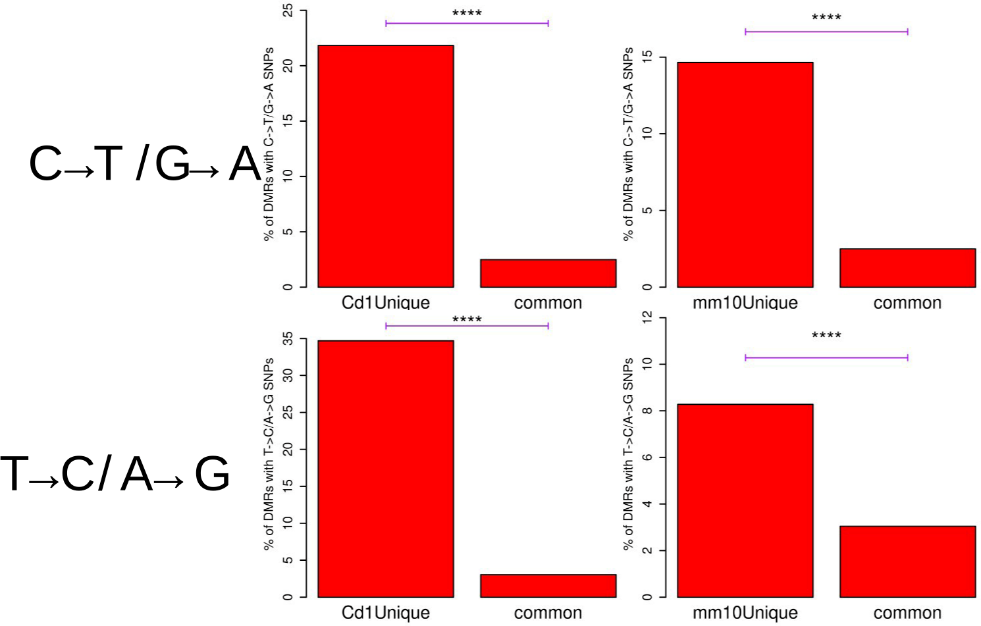
B.**

**Figure S4. Related to Figure 3. A** Barplots showing the percent increase in the number of nonduplicate BS-seq reads mapped when mapping to Cd1 rather than mm10, for the indicated

**Figure S4 (Cont.)**

samples. Positive values indicate more reads mapped in Cd1 than mm10. **B** Barplots comparing the percentage of the indicated DMR sets overlapping with a uniform C->T or G->A SNP (mm10->Cd1; top row), or comparing the indicate DMR sets overlapping with a uniform T->C or A->G SNP (mm10->Cd1; bottom row). Cd1Unique: DMRs that are called when mapping to Cd1 but not when mapping to mm10; mm10Unique: DMRs that are called when mapping to mm10 but not when mapping to Cd1; common: DMRs that are called both when mapping to Cd1 and to mm10. P-values in this figure were calculated by Fisher’s exact test, with cutoffs shown as follows: * p < .01 ; ** p < .001 ; *** p < .00001 ; **** p < .0000000001.

**Figure S5**

**
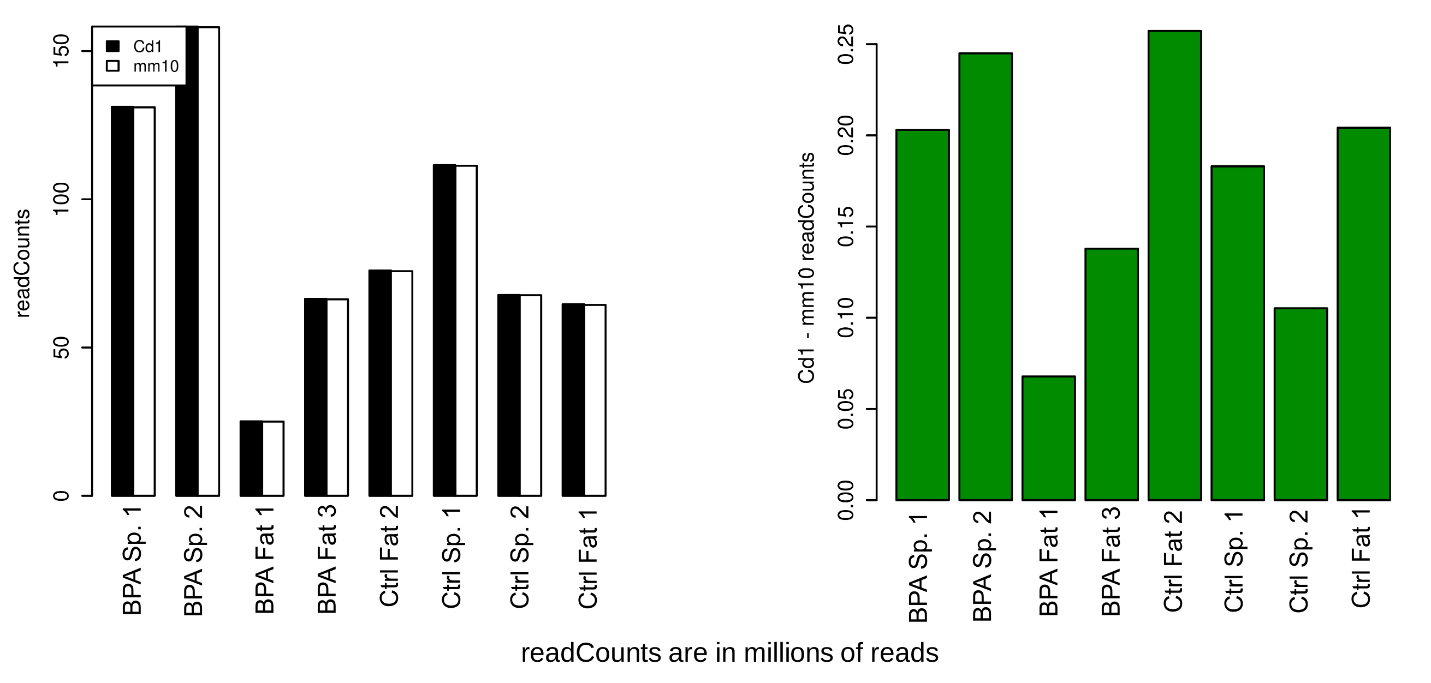
A.**

**B.**

**
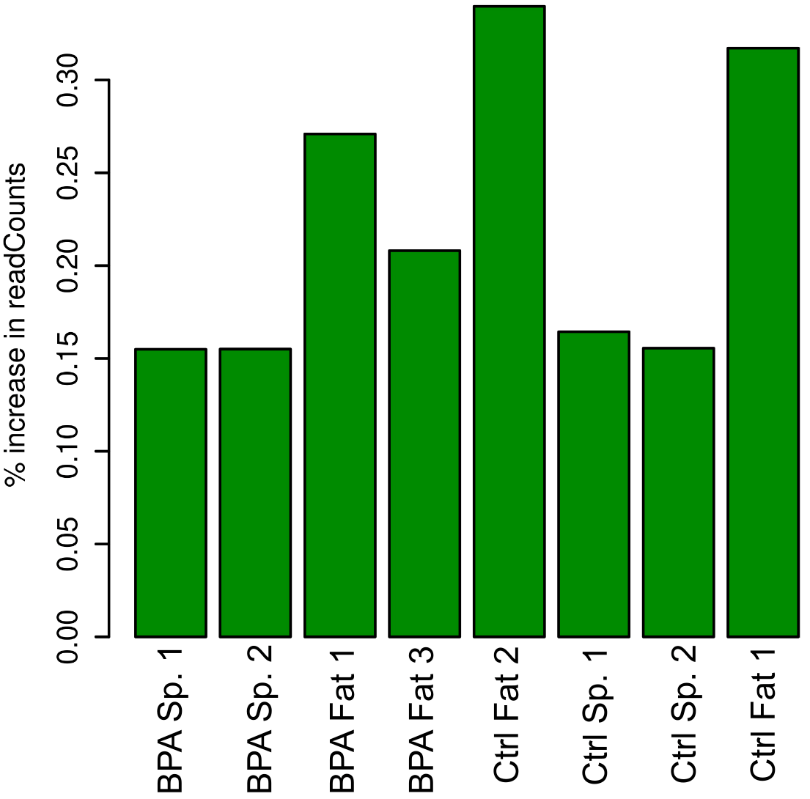
**

**Figure S5. Related to Figure 4. A** Left panel: Barplots showing the number of non-duplicate ATAC-seq reads mapped to either Cd1 or mm10 from the indicated samples. Right panel: Barplots

**Figure S5 (Cont.)**

showing the difference between the number of nonduplicate ATAC-seq reads mapped in Cd1 and mm10. Positive values indicate more reads mapped in Cd1 than mm10. **B** Barplots showing the percent increase in the number of nonduplicate ATAC-seq reads mapped when mapping to Cd1 rather than mm10. Positive values indicate more reads mapped in Cd1 than mm10.
